# Evaluation of the administrative management model in a municipality in Peru, incorporating the Intelligent Organization Theory

**DOI:** 10.12688/f1000research.143937.1

**Published:** 2024-04-30

**Authors:** José Elider Tarrillo Carrasco, José Humberto Becerra Santa Cruz, Carlos Hugo Luna Rioja, José Carloman Saldaña Castillo, Rogger Orlando Morán Santamaría, Francisco Eduardo Cúneo Fernández, Nikolays Pedro Lizana Guevara, Cynthia Vanessa Coronel Benites

**Affiliations:** 1 Universidad Nacional Pedro Ruiz Gallo, Lambayeque, Lambayeque, Peru; 2 Universidad Nacional de Trujillo, Trujillo, La Libertad, Peru; 3 Universidad Nacional Toribio Rodriguez de Mendoza de Amazonas, Chachapoyas, Amazonas, Peru; 4 Universidad Cesar Vallejo, Trujillo, La Libertad, Peru

**Keywords:** Administrative management, intelligent organizations, systemic learning, management models.

## Abstract

**Background:**

This study begins with the analysis of the current management models and their degree of effectiveness in municipal administration. Its aim is to design an administrative management model that enables effective administration in the District Municipality of Nueva Cajamarca, Peru, based on the theory of intelligent organizations.

**Method:**

The research type employed in this study is diagnostic-propositional, utilizing both deductive and inductive methods, in alignment with a mixed-method approach and a non-experimental nature of the study. Data was collected from three distinct populations, including the 189 municipal employees engaged in administrative roles, who were subjected to a 50-question survey. This survey aimed to assess their perceptions regarding the current management model and its relationship with administrative effectiveness. Additionally, interviews were conducted with three experts to gain deeper insights into the behavior of the variables under investigation.

**Results:**

Finally, documentary information about the management models currently in use was collected. This facilitated the triangulation of data collection, processing, analysis, and inferences from three sources of information. The results reveal a positive, direct, and significant correlation between the management model and administrative effectiveness. It becomes evident that the current management model is deficient, resulting in a low level of administrative effectiveness.

**Conclusion:**

The management model based on the theory of intelligent organizations was validated using a rubric by experts in effective management. The main pillars of this model include transformational leadership, structural change, and cultural change.

## Introduction

In a global context, elements such as technological innovation, market globalization, and social changes are causing constant dynamics in the administrative management of any organization, necessitating ongoing adaptation ( Coffay & Bocken, 2023; Kashani et al., 2023; Nasir & Zhang, 2024; Huynh et al., 2023; Del Carpio, et al., 2021). Hence, the need to develop strategies and tools for building organizations that are conducive to learning ( Forliano et al., 2023; Olszak, 2022; Senge et al., 2018). This implies that institutions must transition to new management models rooted in systemic thinking approaches as a foundation for personal growth, mental frameworks, the creation of shared visions, and group learning ( Senge, 2019; Funke et al., 2023; Pedraza et al., 2023; Chiu & Lin, 2022).

On the other hand, an intelligent organization embarks on a process to extract the highest benefit from its experiences. These organizations resemble societies of organizations within modern society and represent complex, multidimensional systems at various levels of human activity ( Chiavenato, 2014; Tian et al., 2022; Capatina et al., 2016). To effectively achieve their objectives and attain success, it is essential to focus on resource and personnel management ( Banmairuroy et al., 2022; Gómez and Balkin, 2003). Furthermore, it is crucial to evolve empirically and adapt proactively, involving both expansion and strategic restraint to establish a strong foundation ( Wang et al., 2023; Erazo, 2013).

However, in these organizations, survival and adaptation are not the sole factors of importance. They must also develop the ability to create and learn, grounding their growth in five core disciplines: systemic thinking, mental models, shared vision, team learning, and personal mastery ( León et al., 2003; Krskova & Breyer, 2023). In this regard, according to Senge (2019), each of these disciplines represents a path of development to acquire competencies and skills, with systemic thinking considered complementary to the others ( Panagiotopoulos et al., 2023).

In Peru, among the areas of application for management models, public administration is of the greatest interest. This is because it has the mission of serving and directly and continually managing the relationships between the government and the citizenry ( Rojas Palacios et al., 2023). In this regard, as stated by Criado (2016), paradigms in public administration are constantly evolving, making it necessary to improve personnel management, teams, resources, the economy, and organizational interrelations ( Reyes & Castañeda, 2020). This need arises from the fact that continuous improvement is the primary objective of intelligent organizations, and it can be applied to public administration to harness and enhance the capacity for learning from each member through a systematic mechanism ( Reyes & Castañeda, 2020).

Furthermore, there is an observed reality of limited progress in the modernization of the state, leading to issues such as deficient designs of organizational functions, inadequate policies and human resource management, the absence of effective planning systems affecting the budget, minimal impact of evaluations on the necessary changes, and a lack of goals and efficient administration.

This reality is reflected in the District Municipality of Nueva Cajamarca, which exhibits an inadequate administrative system due to a lack of managerial competencies ( Visalot, 2016). In this regard, the general question was raised: “What would the administrative management model be to enable the development of the District Municipality of Nueva Cajamarca - Peru, incorporating the theory of intelligent organizations?”

The research provides theoretical utility as it could validate a theory in the field of public management in Peru, serving as fundamental knowledge that can be replicated in all public sector entities in the Peruvian state. It also serves as a reference for future research work involving triangulation designs, new data collection instruments, processing and analysis. Additionally, it offers practical utility by enabling the implementation of a highly effective intelligent management model within the municipality, which would translate into improved governance and territorial governance. This, in turn, would lead to the provision of quality services, a reduction in social disparities, and the creation of public value, ultimately enhancing the quality of life for the population.

## Methods

The research had a descriptive-propositional. In the initial stage, the relationship between the current management model and the administrative effectiveness being implemented by the municipality was evaluated to determine the direction, intensity, and significance between these two variables ( Hernández-Sampieri and Mendoza, 2018). Subsequently, a new municipal management model based on Peter Senge's theory of intelligent organizations is proposed with the aim of enhancing municipal administrative effectiveness.

The study's nature is mixed, involving the triangulation of both quantitative and qualitative information through surveys, interviews, and documentary sources. A correlational design was used for the quantitative part (surveys), and a phenomenological approach was employed for the qualitative aspect (interviews and documents), following a sequential process (first measuring quantitatively and then interpreting to deepen the characterization of the variables).

The research design is non-experimental, as variables were not manipulated; instead, events were analyzed after they occurred ( Carrasco, 2017).

### Population and sample

For the quantitative part of the study, the entire population, known as the census population, consisting of 189 municipal employees in administrative roles, was included. The formula for finite population sampling was not used. As for the qualitative part, based on the author's discretion, three individuals were considered representative of the district (1 representative from the Municipal Workers Union, 1 former mayor, and 1 municipal manager) (Hernández et al., 2014).

### Data collection techniques and instruments

In terms of data collection techniques, instruments, equipment, and materials, three types were used: Surveys, interviews, and evaluation techniques - rubrics.

The survey, conducted using a questionnaire, was employed to measure the perception of municipal employees regarding the type of management model being implemented in their job performance. The survey consisted of 50 items, with 20 items related to the administrative management variable and 30 items related to intelligent organizations.

Interviews are a technique that allows for detailed data collection, as the informant shares information orally with the researcher. This is a valuable tool for data collection ( Fontana and Frey, 2005; Díaz et al., 2013). This is why interviews were used in the research, which served to triangulate the information provided by the interviewees with the data obtained from the survey of municipal employees.

The evaluation rubric was structured, according to its table of contents, into five (5) parts, each with a given number of items: Part 1, 2 items; Part 2, 9 items; Part 3, 3 items; Part 4, 7 items; Part 5, 2 items; making a total of 23 items to be evaluated, and, each one, was evaluated according to ten (10) indicators: clarity, objectivity, timeliness, organization, sufficiency, intentionality, consistency, coherence, relevance and feasibility.

### Validity and reliability

Before the instruments were used, their validity and reliability were assessed.

Reliability: Reliability was assessed using the Cronbach's Alpha coefficient (α). A pilot test was conducted with employees from 7 similar municipalities, including 5 district and 2 provincial ones. The results showed very high reliability for the Administrative Management variable (0.903) and for the Intelligent Organizations variable (0.952) ( Sanchez, 2022; Vara, 2015; Córdova, 2019).

Validity: Validity was assessed through informed opinions of individuals with expertise and authority to provide information, tests, and judgments. Qualitative evaluation by 5 experts concluded that the instrument's validity qualifies as excellent-good, meaning it is validated. As for the quantitative assessment, a statistical analysis was conducted for each item, calculating the Aiken's V coefficient using binary ratings of 0 or 1, resulting in a validity level of 0.98 for each variable ( Escobar and Cuervo, 2008; Walker and Lev, 1953; Sanchez, 2022; Córdova, 2019).

Evaluation Technique with Rubric: The rubric technique was used to theoretically assess the proposed administrative management model through expert judgment. According to Menzala & Ortega (2021), a rubric is an assessment tool to communicate quality expectations. Its validity was assessed through the judgment of five experts, obtaining an Aiken's V coefficient of 1, and its reliability, evaluated through a pilot test, yielded a result of 0.643 according to the KR-20 coefficient, indicating a good level of reliability.

#### Data analysis

For processing and analyzing quantitative data, software such as Excel, SPSS (V. 26), and Atlas.ti were used. The Kolmogorov-Smirnov normality test was employed, as the sample size exceeded 50 data points, and the non-parametric Spearman's Rho correlation coefficient was used, as the significance level was less than 0.05.

Consent

During the conduct of the study, we ensured that the research population was always fully informed. This was achieved through signed informed consent, each participant, a detailed description of the study, its objectives, the procedures involved and any potential impact.

## Ethical approval

The research was approved by “Resolution N°091-2023-EPG” by the “Unidad de TeleEducación de la Escuela de Posgrado de la Universidad Nacional Pedro Ruiz Gallo de Lambayeque”, on January 27, 2023.

## Results

### Description of the current administrative management model and its level of effectiveness

Three management models were evaluated: the 7-S Management Model (M7-S), the Results-Oriented Management Model (GpR), and the Process-Oriented Management Model (GpP). The qualitative description of the current administrative management model was based on information provided by three interviewees and processed in Atlas.ti. It yielded the following categories: intelligent organizations (15 codes), administrative management (5 codes), oversight bodies (7 codes), state structure (7 codes), deficient management (8 codes), planning area (5 codes), and deficient vision (7 codes), all of which are interconnected and coordinated. The category of intelligent organizations accumulates the highest number of codes (15), indicating an interest in their implementation but also concern about rating the current management as deficient (8 codes).

The quantitative description of the current administrative management model shows perceptions from the sample. 55%, 53%, and 38% of respondents state that actions aligning with the M7-S, GpR, and GpP models are always or nearly always implemented, respectively. Meanwhile, 45%, 47%, and 62% report that these actions are only sometimes, rarely, or never implemented. The M7-S model is the most prevalent, while the GpP model is the least applied. However, the implementation of all three models is limited, hindering the development of comprehensive management and the introduction of tools that ensure a positive impact on municipal activities and the quality of life for citizens. In other words, the pursuit of continuous improvement in activities is not being effectively pursued, with a maximum of 13% implementing it consistently.

**Figure 1.**
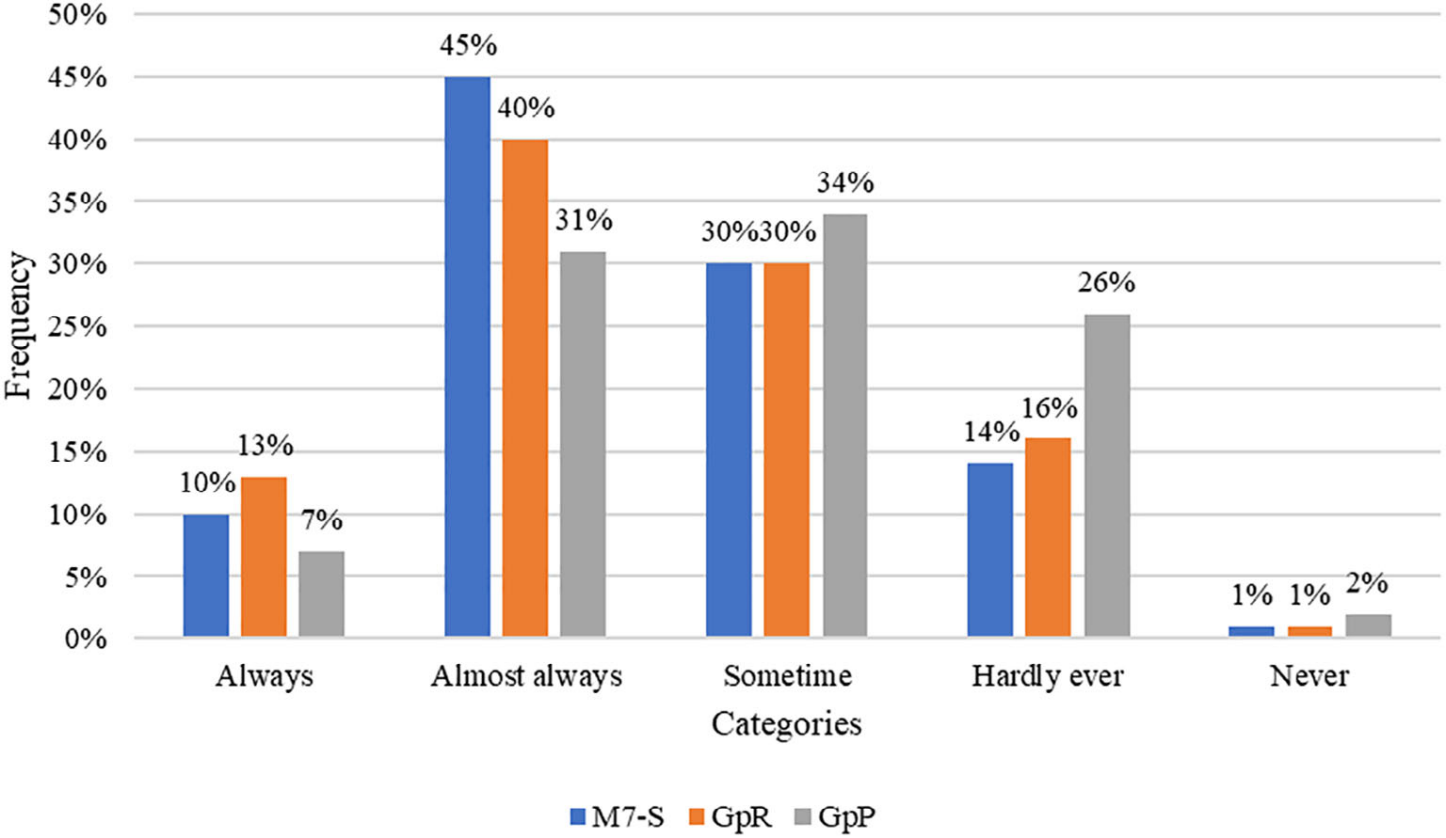
Results of the management models, as per the workers' perception.

To establish the level of effectiveness of the current management models, following Delgado & Marcos (2018) and Vásquez & Farje (2020), categories were instituted for each dimension: efficient (5), good (4), fair (3), poor (2), and deficient (1), resulting in an average of 10.17%, indicating “Efficient.” The results showed that the Results-Oriented Management model prevails with a percentage of 13.43%. On average, 38.89% falls into the “Good” category, with the 7-S model prevailing at 45.28%. On average, the categories “Fair,” “Poor,” and “Deficient” obtained percentages of 31.61%, 18.28%, and 1.04%, respectively. In this context, the current management model would be rated as having a “Fair" level of effectiveness, which does not ensure efficient administrative management. Therefore, it is necessary to propose a management model that incorporates systemic thinking as the foundation of intelligent organizations to improve this situation ( Figure 1).

Qualitatively, the interviewees expressed that regarding the application of the Process-Oriented Management model (GpP), it is limited, but it seeks to enhance the quality of meeting needs, as measured by service management indicators. They are unfamiliar with ISO 9000 standards that would enable them to ensure quality, and the process map is not designed, resulting in poor management and weaknesses in continuous improvement planning.

### Requirements demanded by the implementation of an administrative management model in the era of intelligent organizations

Based on the obtained data, descriptive analyses, normality tests, and correlation between variables were conducted to determine the requirements needed to optimize municipal management by proposing the implementation of an administration model based on the theory of intelligent organizations. In this context, the results are presented as follows:

Regarding descriptive statistics, the mean, standard deviation (SD), skewness, and kurtosis of the intelligent organization’s variable are shown. The mean of 101.57 represents the average score obtained, considering that a Likert scale has been assigned values from 1 to 5. This implies that the minimum score to obtain would be 30 (if all items received a value of 1), and the maximum would be 150 (if all items received a value of 5). Therefore, the mean (101.57) indicates that the average scores fall between 3 and 4, meaning between “almost always" and “sometimes."

The normality test results suggest that the data distribution is not normal (p < 0.05). Therefore, the non-parametric statistical test Rho of Spearman was used.

The analysis also examined the relationship between administrative management and intelligent organizations, resulting in a correlation coefficient of 0.672 (p < 0.01), indicating a strong and positive direct relationship.

From the qualitative analysis, it is evident that the application of a municipal administrative management model based on intelligent organizations is feasible. There is willingness, but it is clear that the structure must first be adjusted to prioritize technical-political commitment in order to provide quality services. To achieve this, the following conditions must be met: having an appropriate structure, an organizational climate tailored to needs, suitable office equipment, strengthening and promoting professional development, fostering a culture of attitudinal change, depoliticizing policies, guidelines, and strategies, and implementing salary improvements.

### Evaluation of the development of learning in personal mastery, mental models, shared vision, team learning, and systems thinking within the institution

It is observed that 52%, 47%, 49%, 49%, and 46% of the surveyed individuals reported that actions consistent with the disciplines of personal mastery (DomPer), mental models (ModMen), shared vision (VisComp), team learning (AprendEq), and systems thinking (PensSist) are always or almost always implemented in administrative management. On the other hand, 48%, 53%, 51%, 51%, and 54% indicated that these actions are only performed sometimes, rarely, or never. Personal mastery is the most emphasized dimension, while systems thinking is the least emphasized.

From the results analysis, it appears that there is little commitment among municipal employees regarding personal mastery. As for mental models, there is a need to work on improving positive aspects such as paradigms, beliefs, maps, images, assumptions, and more. This suggests that the percentage of those who share the institutional vision is low, as is the case with team learning. All of this leads to a limited connection between the various components of the organizational system to achieve desired results, with only 9% of municipal employees consistently practicing systems thinking.

**Figure 2.**
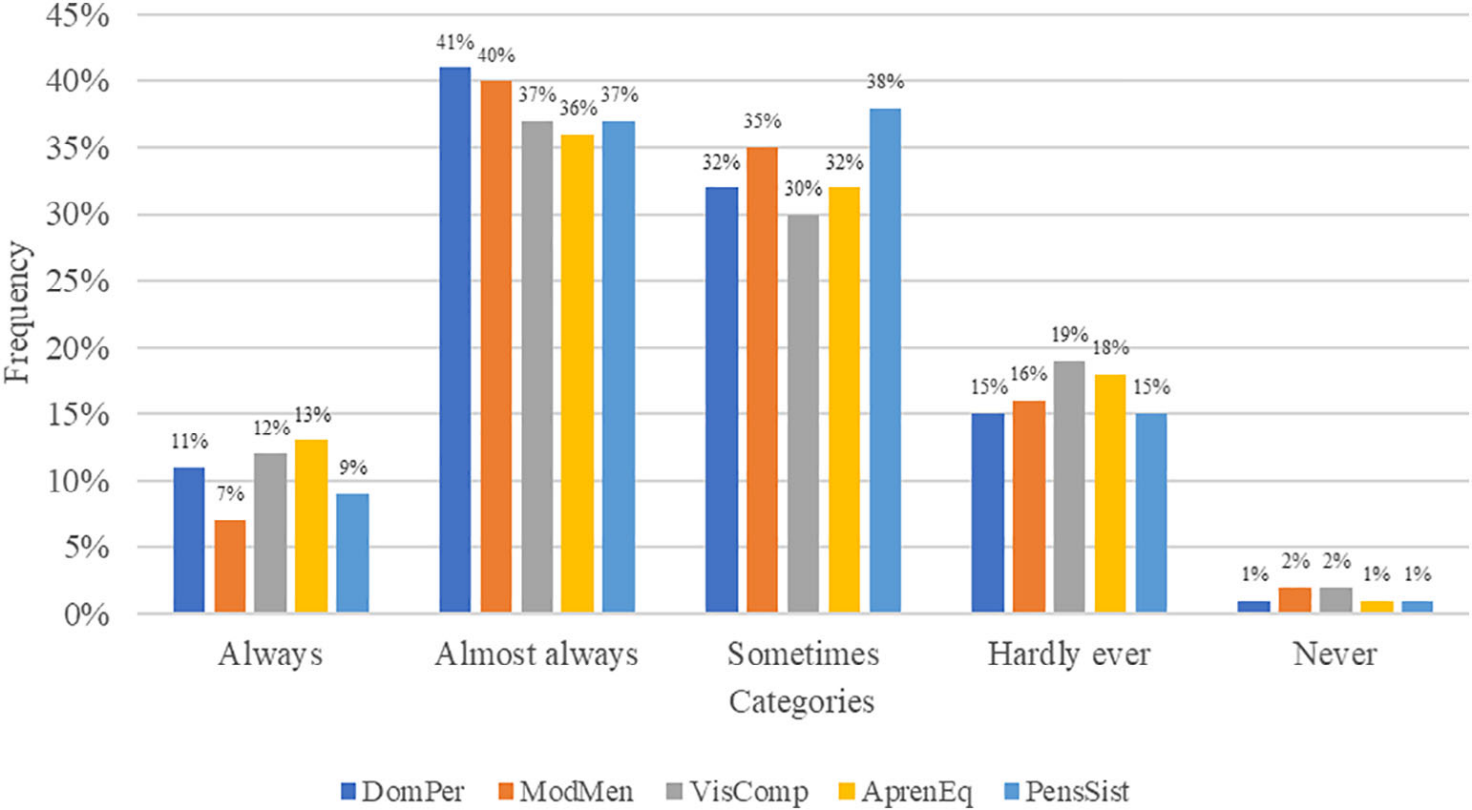
Management under the dimensions of intelligent organizations.

The information presented was triangulated with the data acquired from the interviewees. Regarding personal mastery, they indicate that the workers lack identity and relevance, are dissatisfied, demotivated, and lack professionals with the required profiles. Concerning mental models, they express that these are generally based on personal perspectives rather than institutional ones, which is why institutional objectives and vision are not adequately shared or socialized. Team learning is primarily facilitated by leaders and is not extended to all employees, and they agree that the practice of systems thinking as a behavioral pattern aimed at learning and continuous improvement is still in its early stages ( Figure 2).

### Development of the structure of a municipal administrative management model based on the theory of intelligent organizations

The proposed model, named “Administrative Management Model for Developing an Intelligent Organization in the District Municipality of Nueva Cajamarca - Peru," is structured into five parts: model definition, model disciplines and continuous improvement proposal, management theories and their relation to the model, considerations for model implementation, and considerations for model evaluation. Given that in Peru, public sector organizations struggle with governance at different levels of government, according to Chanamé (2017), these difficulties generally include a deficient planning system, government failure to collect people's needs, inefficient organizational and functional structure, inadequate production of goods and services, and weak governmental and interinstitutional coordination.

Starting with a baseline description of each discipline:

Personal Mastery (DomPer), as an individual discipline of growth and learning, allows for an objective observation of the context and linking work to individual and group learning. In this regard, 48% of municipal personnel state that they sometimes, rarely, or never receive actions to strengthen personal mastery. This perception aligns with the information obtained from the interviewees.

Mental Models (ModMen), as an individual discipline of permanent mechanisms, its practice allows for perceiving the real world from different perspectives. In this sense, 53% of municipal personnel state that they sometimes, rarely, or never receive actions to strengthen mental models. This perception aligns with the interviewees' responses, who indicate that the employees' performance is based on personal perspectives rather than institutional ones.

Shared Vision (VisComp), as a group and systemic discipline that proposes appropriate transformations in the real world. The obtained perception indicates that 51% of municipal personnel sometimes, rarely, or never receive actions to strengthen it. This aligns with the interviewees' opinions, who state that the socialization of the institutional vision and objectives is deficient, and only a small percentage is oriented towards implementing an intelligent organization due to employees' lack of knowledge.

Team Learning (AprendEq), as a group discipline, consists of the process of aligning and developing the group's ability to generate ideas, innovate, and improve the quality of desired results. It is reported that 51% of municipal personnel sometimes, rarely, or never receive actions to strengthen this type of learning. The interviewees indicate that this learning is primarily facilitated by leaders, referring to those in offices with greater responsibility for executing established tasks.

Systemic Thinking (PensSist), as a discipline that combines others, 54% of municipal personnel state that they sometimes, rarely, or never receive actions to strengthen this discipline. This data is consistent with the interviewees' opinions, who state that behavioral patterns towards learning and continuous improvement are in their early stages.

**Figure 3.**
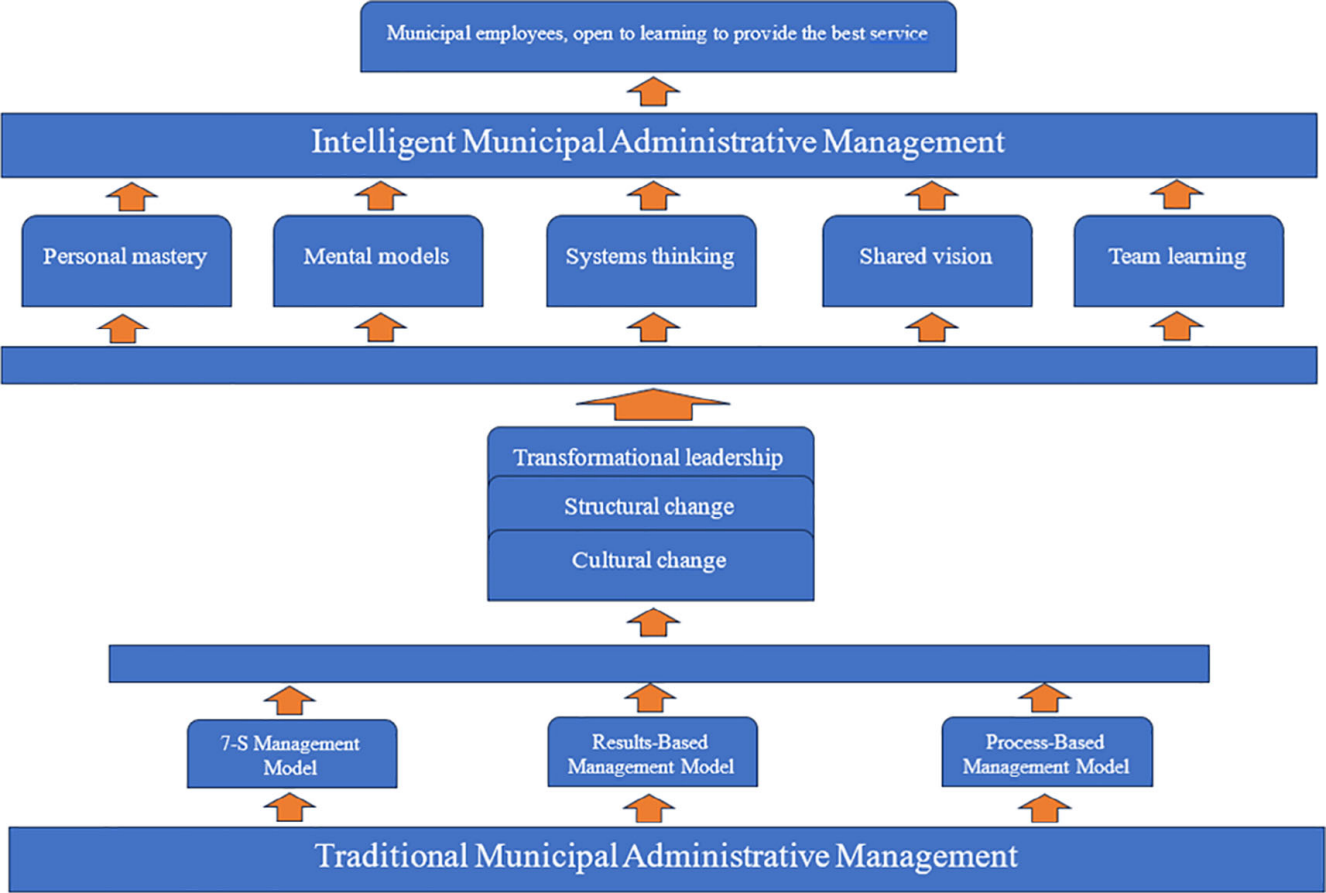
Intelligent Municipal Administrative Management Model.

From the information presented for each of the disciplines, it is evident that there is a significant gap that demands the design of competency development strategies for municipal employees to set the institution on the path of intelligent organizations. Based on this, the implementation of strategies for developing the profile of municipal human talent is proposed. The proposal for continuous improvement is composed of three components: knowing how (knowledge and skills), being able to do (procedural), and wanting to do (attitudinal). The baseline is described based on the presented information, and the expected achievement level is projected at the end of the model's implementation. To execute the competency proposals for continuous improvement, three fundamental aspects are considered, namely transformational leadership, structural change, and cultural change, and the disciplines of intelligent organizations are applied to have municipal collaborators open to learning to provide the best service to the user population ( Figure 3).

Interviewees, regarding skills development for implementing strategies, state that it is a legal obligation, implemented in strategic areas as needed. However, information systems, production processes, control, and budgets are limited and outdated. Nevertheless, they provide transparent and timely information because they are supervised by control bodies. On the other hand, promotion is occasional, and the leadership style has a deficient vision. People are moderately oriented towards achieving objectives since they are not shared, leading to inefficient personnel.

To transform the Municipality of Nueva Cajamarca into an intelligent organization, it is necessary for the theory of transformational leadership to be applied at all levels, understanding that leaders are not necessarily only those in high positions within an entity, but that any employee can be a leader from their workspace. Leadership is the ability to mobilize people towards shared goals and objectives ( Fischman, 2017). According to Hermosilla et al. (2016), transformational leadership is considered one of the most appropriate leadership styles for driving processes of change and innovation in the organization.

Subsequently, a structural change is needed, which, according to Yoguel (2014), is defined as a process of qualitative and quantitative transformation that occurs in a particular production structure.

Lastly, the most important aspect is cultural change, which, according to Taylor (1995), allows understanding the nature of culture as a necessary element to comprehend human behavior, as presented in the model of intelligent municipal administrative management.

## Discussion

The discussion begins by accepting the hypothesis that incorporating an administrative management model based on intelligent organizations facilitates institutional development. Romera (2016) indicates that knowledge, skills, and competencies must be continuously improved. Therefore, when describing the current administrative management, the 7-S model by McKinsey Company in 2012 was analyzed. The results showed that only 10% of employees always apply this model, and 1% never do. This is in line with Peralta (2017), who concludes that the functions of local governments are limited and do not strengthen decentralization. García & Mendoza (2018) indicate that the strategy, personnel, systems, and structure are unfavorable, while leadership styles and shared values received favorable scores. However, Ortiz (2020) disagrees and states that at UPLA-Huancayo, 77% agree, and only 17% disagree.

Management based on the results-oriented management model, which, according to Makon (2017), allows the management and evaluation of the performance of state organizations in relation to public policy, is applied by only 13%, and 1% never apply it. This aligns with Ccoa (2018), who indicates that it is not being applied or encouraged, while Cabrejos (2019) maintains that the model's application in the Municipality of San Jose has an average index of 1.56. However, this contradicts Shack & Rivera (2017) and Esparta & Paco (2017), who state that it has a positive impact on performance-based budgeting. Additionally, Bernuy (2017) asserts that 81% of the budget is aligned with results-focused programs.

The process-oriented management model, as mentioned by Aranda et al. (2018), is felt to be an obligation for the state to fulfill its commitments. However, it is not progressing at the required pace, as only 7% always apply it, and 2% never do. This is consistent with Aranda, Ordóñez, and Peralta (2018), who identified that level 0 processes in the Ministry of Agriculture are not being implemented and recommend implementing process management. Begazo & Fernandez (2017) affirm that public administration should work with process management.

In terms of the demand for intelligent organizations management models, Senge (2019) points out that it requires a constant commitment to learning. The results in individual dimensions, personal mastery and mental models, have a positive Pearson correlation of 0.723, in line with Rojas (2018) who indicates that the key to becoming an intelligent organization is to implement a business philosophy with values and employee involvement in decision-making. Martínez et al. (2017) state that intelligence and knowledge are intangible assets for the organization. Regarding the collective dimensions, systems thinking is related to shared vision, and team learning has correlation indexes of 0.639 and 0.623, respectively. These results are consistent with Lozano & Gonzalez (2016), who present a comprehensive view of the changes needed for the development of organizational intelligence. In this regard, Seminario & Seminario (2020) concluded that in intelligent organizations, achieving team learning enhances competitiveness.

Furthermore, the development of learning in intelligent organizations' disciplines was evaluated. In terms of personal mastery, 11% always apply it, 41% almost always, 32% sometimes, 15% almost never, and 1% never. For mental models, 7% always practice it, 40% almost always, 35% sometimes, 16% almost never, and 2% never. This aligns with Quiroz (2017), who indicates that although the organization theoretically exhibits plans and proposals for intelligent management, in practice, it falls short of being so. However, it disagrees with Barrena (2017), who concludes that 95% of workers consider a high degree of personal mastery, and 85% consider the socialization aspect to be moderate. In terms of shared vision disciplines, 12% always apply it, 37% almost always, 30% sometimes, 19% almost never, and 2% never. For team learning, 13% always put it into practice, 36% almost always, 32% sometimes, 17% almost never, and 1% never. Finally, for systems thinking, 9% always apply it, 37% almost always, 39% sometimes, 15% almost never, and 1% never. These results are in line with López et al. (2016), Passailaigue et al. (2017), and Luza (2017), who indicate a direct link between the learning capacity and strategic decisions.

In the development of the structure of the municipal administrative management model based on the theory of intelligent organizations, the results show that only 10% always apply it, 38% almost always, 34% sometimes, 16% almost never, and 2% never. It is evident that in the criteria expected to always and almost always, they do not exceed the optimal level's average. This aligns with Guevara (2017), who indicates that the organizational climate in the Municipalidad Provincial de Chota is inadequate. Cutipa (2017) states that the organizational level is not coordinated with all workers, consistent with Arroyo (2016) and Rincón et al. (2016), who argue that changing old prevailing schemes is necessary to give way to new management by shifting from bureaucratic models to intelligent organizations.

In summary, this research contributes to the scientific community by introducing a new model of municipal administrative management based on the theory of intelligent organizations. This model will serve as a theoretical foundation for quasi-experimental application and scientific effectiveness testing, aiming to incorporate soft skills into functional tasks to achieve cultural and structural change through transformational leadership. Additionally, it provides validated instruments such as a survey questionnaire, interview questionnaire, and evaluation rubric. Finally, it demonstrates the existence of a significant correlation between municipal administrative management and intelligent organizations, thus supporting the research hypothesis.

## Conclusions

According to the research results, the study provides valuable contributions to science by designing a municipal administrative management model based on intelligent organizations. This model incorporates soft skills to achieve cultural and structural change through transformational leadership. The study also includes validated instruments such as a survey questionnaire, interview questionnaire, and evaluation rubric. Furthermore, it confirms the existence of a significant correlation between variables, thereby supporting the research hypothesis.

In the diagnostic phase, the findings revealed a positive, direct, and significant correlation (0.672, p<0.01) between the variables related to the research problem, general objective, and hypothesis, which is the model of management and administrative effectiveness. It was also demonstrated that the implementation of the dimensions is being applied moderately. Consequently, this study presents a proposal aimed at contributing to a new intelligent management model with both theoretical and practical implications.

The level of perceived administrative management effectiveness at the District Municipality of Nueva Cajamarca is considered moderate. Approximately 51% of the respondents mentioned that actions aligned with the 7-S management models, results-based management, and process-based management are implemented sometimes, almost never, or never. This perception aligns with the statements made by the interviewees regarding the moderate application of these models.

The results of the evaluation of learning development as an intelligent organization indicate that 52% of respondents working at the District Municipality of Nueva Cajamarca mentioned that actions aligned with the dimensions of personal mastery, mental models, shared vision, team learning, and systems thinking are sometimes, almost never, or never implemented. This perception is consistent with the input provided by the interviewees. Therefore, strategies need to be developed through an administrative management model that incorporates dimensions from the intelligent organizations' theoretical approach to improve the performance of municipal workers.

The municipal administrative management model for developing an intelligent organization in the District Municipality of Nueva Cajamarca, Peru, was designed and theoretically validated through expert judgment. An Aiken's content and construct validity coefficient of 0.98 was obtained, indicating a very acceptable level of content and construct. This data underscores the importance of considering these proposed considerations for an effective transition from traditional municipal administration to intelligent municipal administration, achieved through transformational leadership, structural changes, and cultural shifts.

### Ethics and consent

The research was approved by “Resolution N°091-2023-EPG" by the “Unidad de Tele Educación de la Escuela de Posgrado de la Universidad Nacional Pedro Ruiz Gallo de Lambayeque”, on January 27, 2023.

During the conduct of the study, we ensured that the research population was always fully informed. This was achieved through signed informed consent, each participant, a detailed description of the study, its objectives, the procedures involved and any potential impact.

## Data Availability

Zenodo. Evaluation of the administrative management model in a municipality in Peru, incorporating the Intelligent Organization Theory. https://doi.org/10.5281/zenodo.10872138 ( Tarrillo et al., 2024). This project contains the following underlying data:
• COREQ_checklist.pdf • Data.xlsx •
Figure 1. Results of the management models, as per the workers' perception.png •
Figure 2. Management under the dimensions of intelligent organizations.png •
Figure 3. Intelligent Municipal Administrative Management Model.png • Informed Consent.pdf • INSTRUMENTS.pdf Creative Commons Zero v1.0 Universal (CC0 License)

## References

[ref819] Aguilar I Pereda M Mera C : Applying Business Process Modeling to improve IT Incident Management Processes in a Public Entity in Peru. *Journal of Software and Systems Development.* 2020;2020:1–20. 10.5171/2020.109641

[ref2] Aranda M Ordoñez L Peralta C : La gestión por procesos como medio para mejorar la eficacia en el cumplimiento de objetivos institucionales del Minagri.

[ref3] Arroyo J : Gestión municipal desde la perspectiva de las organizaciones inteligentes y ámbito jurídico. *Investigaciones Originales.* 2016;18(2):127–141.

[ref801] Banmairuroy W Kritjaroen T Homsombat W : The effect of knowledge-oriented leadership and human resource development on sustainable competitive advantage through organizational innovation’s component factors: Evidence from Thailand’s new Scurve industries. Asia Pacific Management Review. 2022;27(3):200–209. 10.1016/j.apmrv.2021.09.001

[ref4] Barrena J : Gestión del conocimiento y organización inteligente en la Gerencia de Investigaciones Aduaneras de la SUNAT, Callao – 2016. 2017.

[ref5] Begazo J Fernandez W : Gestión por procesos y su relación con el plan estratégico en un contexto de modernización de la gestión pública peruana. *Gestión En El Tercer Milenio.* 2017;19(37):25–30. 10.15381/gtm.v19i37.13773

[ref6] Bernuy Y : Gestión del presupuesto por resultados de la municipalidad distrital y calidad de vida en los servicios básicos de la población del distrito de Pampas Grande,Ancash, periodo 2014-2016. 2017.

[ref12] Córdova I : *Instrumentos de investigación.* San Marcos;2019.

[ref7] Cabrejos E : Gestión por resultados y capacidad institucional en la municipalidad distrital de San José, Lambayeque. 2019.

[ref802] Capatina A Bleoju G Matos F : Digital transformation in assetintensive organisations: The light and the dark side. Journal of Innovation & Knowledge& Knowledge. 2016;1:144–155. 10.1016/j.jik.2016.01.016

[ref8] Carrasco S : *Metodología de la investigación.* San Marcos;2017.

[ref9] Ccoa G : Análisis de la gestión por resultados de la capacitación en actividades empresariales de la municipalidad distrital de Socabaya en el primer semestre 2018. 2018.

[ref10] Chanamé C : *Deficiencias de la gestión pública en Perú que se deben resolver.* Continental;2017.

[ref11] Chiavenato I : *Teoría general de la administración.* McGrawHill;2014.

[ref803] Chiu M Lin C : Human capital and organizational performance: A moderation study through innovative leadership Attia. Journal of Innovation & Knowledge. 2022;7(4): 100264. 10.1016/j.jik.2022.100264

[ref804] Coffay M Bocken N : Sustainable by design: An organizational design tool for sustainable business model innovation. Journal of Cleaner Production. 2023;427(September):139294. 10.1016/j.jclepro.2023.139294,

[ref13] Criado I : Las administraciones públicas en la era del gobierno abierto. Gobernanza inteligente para un cambio de paradigma en la gestión pública. *Revista de Estudios Políticos.* 2016;173:245–275. 10.18042/cepc/rep.173.07

[ref14] Cutipa M : Las organizaciones inteligentes y la gestión del talento humano en empresas ejecutoras de las obras del programa de mejoramiento y ampliación de servicios de agua y saneamiento en la provincia de Puno, periodos 2015 y 2016. 2017.

[ref17] Díaz L Torruco U Martínez M : La entrevista, recurso flexible y dinámico. Investigación en educación médica. 2013;162–167.

[ref15] Del Carpio H Del Carpio P García F : Validez de instrumento: percepción del aprendizaje virtual durante la CoVId-19. *Campus Virtuales.* 2021;10(2):111–125.

[ref16] Delgado E Marcos R : Efectividad organizacional y gestión administrativa de los docentes de una Institución Educativa, Ica-2018. 2018.

[ref18] Erazo O : *El mentor en las pequeñas organizaciones inteligentes.* Trujillo, Perú: Universidad Nacional de Trujillo;2013. (Tesis Doctoral).

[ref19] Escobar J Cuervo A : *Validez de contenido y juicio de expertos: Una aproximación a su utilización.* ResearchGate;2008;27–36.

[ref20] Esparta K Paco K : El proceso de gestión y el presupuesto participativo por resultados del distrito de San Antonio. *Cañete.* 2017;2015.

[ref21] Fischman D : *El líder transformador 2.* Planeta;2017.

[ref22] Fontana A Frey J : The Interview, from neutral stance to political involvement. *The Sage Handbook of Qualitative Research.* 2005;695–727.

[ref805] Forliano C Bullini Orlandi L Zardini A : Technological orientation and organizational resilience to Covid-19: The mediating role of strategy’s digital maturity. Technological Forecasting and Social Change. 2023;188(June 2022):122288. 10.1016/j.techfore.2022.122288 36590467 PMC9794488

[ref806] Funke A Wilden R Gudergan S : Only senior managers lead business model innovation, or do they? Levels of management and dynamic capability deployment. Industrial Marketing Management. 2023;114(August):181–195. 10.1016/j.indmarman.2023.08.011

[ref23] García H Mendoza J : Diagnóstico organizacional basado en el modelo de las 7’s de Mckinsey en la empresa inversiones Muchik S.A.C. en la ciudad de Mochumí -Lambayeque. 2018.

[ref24] Gómez M Balkin D : *Administración.* Mc Graw Hill;2003.

[ref25] Guevara S : Plan de mejora basado en organizaciones inteligentes para fortalecer el clima organizacional en la municipalidad provincial de Chota - 2016. 2017.

[ref26] Hermosilla D Silvia da Costa AA Páez D : El Liderazgo transformacional en las organizaciones: variables mediadoras y consecuencias a largo plazo. Journal of Work and Organizational. *Psychology.* 2016;32:135–143. 10.1016/j.rpto.2016.06.003

[ref27] Hernández-Sampieri R Mendoza C : *Metodología de la investigación.* McGrawHill;2018.

[ref807] Huynh TN Van Nguyen P Nguyen QN : Technology innovation, technology complexity, and co-creation effects on organizational performance: The role of government influence and cocreation. Journal of Open Innovation: Technology, Market, and Complexity. 2023;9(4):100150. 10.1016/j.joitmc.2023.100150

[ref808] Kashani ES Naeini AB Gholizadeh H : Innovation systems and global value chains: A Cocitation analysis of established linkages and possible future trends. International Journal of Innovation Studies. 2023;7(1):68–86. 10.1016/j.ijis.2022.09.003

[ref809] Krskova H Breyer YA : The influence of growth mindset, discipline, flow and creativity on innovation: Introducing the M.D.F.C. model of innovation. Heliyon. 2023;9(3):e13884. 10.1016/j.heliyon.2023.e13884 36873469 PMC9982030

[ref28] León M Tejada G Yataco M : *Las organizaciones Inteligentes.* Industrial Data;2003;82–87.

[ref29] López E García F García S : Atributos de la organización que aprende: una revisión de la literatura. *Revista Internacional De Organizaciones.* 2016;16:59–81. 10.17345/rio16.59-81

[ref30] Lozano J Gonzalez C : Desarrollo de un modelo de gestión de la inteligencia organizacional para la compañía Gráficas Modernas S.A. *Universidad & Empresa.* 2016;17(29):1–10.

[ref31] Luza A : Estilos de gestión en el aprendizaje organizacional de la Institución Educativa Parroquial Nuestra Señora de Copacabana, Rímac-2017. 2017.

[ref32] Makon MP : *Políticas presupuestarias y gestión por resultados.* CLAD;2017.

[ref33] Martínez M Gómez H Martínez J : La gestión de la incertidumbre: empresas inteligentes con trabajadores del conocimiento. 2017;6(8):132–143.

[ref34] Menzala C Ortega E : Rúbrica como instrumento de evaluación en educación superior. Dominio de las ciencias. 2021;1020–1034.

[ref810] Nasir MH Zhang S : *Innovation and Green Development Evaluating innovative factors of the global innovation index: A panel data approach.* 2024;3(August 2023).

[ref811] Olszak CM : Business Intelligence Systems for Innovative Development of Organizations. Procedia Computer Science. 2022;207:1754–1762. 10.1016/j.procs.2022.09.233

[ref35] Ortiz A : El Modelo de las 7-S de Mckinsey en la Gestión administrativa en el local central de la UPLA-Huancayo. 2020.

[ref812] Panagiotopoulos P Protogerou A Caloghirou Y : Dynamic capabilities and ICT utilization in public organizations: An Empirical testing in local government. Long Range Planning. 2023;56(1):102251. 10.1016/j.lrp.2022.102251

[ref37] Passailaigue M Márquez F Ortega C : Bases de una estrategia de gestión del conocimiento para la universidad inteligente de clase mundial. *Espacios.* 2017;38:1–13.

[ref813] Pedraza-Rodriguez JA Ruiz-Velez A Sanchez-Rodriguez MI : Management skills and organizational culture as sources of innovation for firms in peripheral regions. Technological Forecasting and Social Change. 2023;191(April2022):122518. 10.1016/j.techfore.2023.12251

[ref818] Peralta H : *Level of empowerment of local governments in their health functions in the process of decentralisation - Arequipa of decentralisation - Arequipa - 2017.* Arequipa: Universidad Nacional San Agustin;2017.

[ref38] Quiroz M : Caracterización del proceso de aprendizaje organizacional en United Airlines, Chile. Perspectiva de los trabajadores. 2017.

[ref814] Reyes M Castaneda P : Aplicacion del Modelo de Aceptacion Tecnologica en Sistemas de Informacion de la Administracion Publica del Peru. *Revista Peruana de Computacion y Sistemas.* 2020;3(1):15–22. 10.15381/rpcs.v3i1.18350

[ref39] Rincón Y Contreras J Prieto R : Comunicación como elemento clave para afrontar el cambio en las organizaciones inteligentes. *Encuentro de investigación ASCOLFA.* 2016;2016:1–18.

[ref40] Rojas J : Capital humano: un desglose teórico para su operatividad en organizaciones inteligentes. *CICAG: Revista del Centro de Investigación de Ciencias Administrativas y Gerenciales.* 2018;16(1):43–54.

[ref815] Rojas Palacios LE Arbulu Perez Vargas CG Reyes Perez MD : Digital Government Management Model for the Modernization of Electronic Services in a Municipality. Peru Case Communications in Computer and Information Science. 2023; Vol.1835, pp.254–261. Springer Science and Business Media Deutschland GmbH. 10.1007/978-3-031-36001-5_33

[ref41] Romera F : Estudio sobre las organizaciones inteligentes en Andalucía. *Revista Fuentes.* 2016;18(1):15–32. 10.12795/revistafuentes.2016.18.1.01

[ref42] Sanchez F : *El Instrumento y su estadística en una tesis.* Centrum Legalis;2022.

[ref43] Seminario R Seminario R : La organización inteligente: una mirada hacia la estabilidad empresarial. *Innova Sciences Negocios.* 2020;1(3):57–66.

[ref44] Senge P : *La quinta disciplina.* Argentina: Granica;2019.

[ref45] Senge P Charlotte R Ross R : *La quinta disciplina en la práctica. Estrategias y herramientas para construir la organización abierta al aprendizaje.* GRANICA;2018.

[ref46] Shack N Rivera R : Seis años de la gestión por resultados en el Perú. 2017.

[ref36] Tarrillo J Becerra J Luna C : Evaluation of the administrative management model in a municipality in Peru, incorporating the Intelligent Organization Theory.[Data set]. *Zenodo.* 2024. 10.12688/f1000research.143937.1 PMC11452770 39371550

[ref47] Taylor F : *Principios de la Administración Científica.* Herrero Hnos. S. A;1995.

[ref816] Tian H Li Y Zhang Y : Digital and intelligent empowerment: Can big data capability drive green process innovation of manufacturing enterprises? Journal of Cleaner Production. 2022;377(August):134261. 10.1016/j.jclepro.2022.134261

[ref49] Vásquez K Farje JD : Efectividad de la gestión administrativa en los gobiernos locales altoandinos, Luya, región Amazonas. *Revista Científica UNTRM.* 2020;3(3):60–66.

[ref48] Vara A : 7 pasos para elaborar una tesis. Macro. 2015.

[ref50] Visalot M : Percepción de la gestión administrativa y su relación con la cultura tributaria, en la Municipalidad Distrital de Nueva Cajamarca, Año 2016. 2016.

[ref51] Walker H Lev J : Análisis de variación. En H. Walker, & J. Lev, Inferencia estadística. 1953.

[ref817] Wang X Liu Z Li J Lei X : How organizational unlearning leverages digital process innovation to improve performance: The moderating effects of smart technologies and environmental turbulence. Technology in Society. 2023;75(April):102395. 10.1016/j.techsoc.2023.102395

[ref52] Yoguel G : ¿De qué hablamos cuando hablamos de cambio estructural? Una perspectiva evolucionista-neoschumpeteriana. *La estructura productiva argentina. Evolución reciente y perspectivas. Buenos Aires.* Argentina: CEPAL;1-3 de Octubre de 2014.

