# Supplementary material for: Evaluation of the administrative management model in a municipality in Peru, incorporating the Intelligent Organization Theory
Source: F1000Res. 2024 Apr 30;13:425. [Version 1] doi: 10.12688/f1000research.143937.1 (PMC11452770; doi:10.12688/f1000research.143937.1)
Supplement: Peer review history

# Reviewer report: Reviewer response for version 1

**DOI:** 10.5256/f1000research.157664.r303379

**Published:** 2024-10-04

**Authors:** Hugo Espinoza Rodríguez

**Affiliations:** 1 Universidad Senor de Sipan, Chiclayo, Lambayeque, Peru

The study focuses on evaluating the current administrative management models in a Peruvian municipality and proposes a new model based on the theory of intelligent organisations.

 The presentation on the research purpose demonstrates a well-executed systematic review of high-impact databases, which makes the information highly useful for the development of this research.

 The theories put forward in the research align with the study’s purpose, and the methodology is highly appropriate for collecting both quantitative and qualitative data.

 In the context of Peruvian municipalities and their deficiencies in service quality, this research contributes by proposing a new management model based on the theory of intelligent organisations, aiming to improve municipal workers' performance and transform the municipality into an intelligent organisation.

 The conclusions are consistent with the research, highlighting a solid proposal to provide intelligent solutions within the organisations under study.

 As a reviewer, the evaluation is acceptable, and it is certain that this study will serve as a valuable precedent for significant projects in municipalities and/or organisations. It is worth mentioning that the application of circular economy principles would contribute greatly to the reuse of materials, the creation of more sustainable management systems, and the reduction of waste through recycling and composting, as well as renewable energies.

Is the work clearly and accurately presented and does it cite the current literature?

Yes

If applicable, is the statistical analysis and its interpretation appropriate?

Yes

Are all the source data underlying the results available to ensure full reproducibility?

Yes

Is the study design appropriate and is the work technically sound?

Yes

Are the conclusions drawn adequately supported by the results?

Yes

Are sufficient details of methods and analysis provided to allow replication by others?

Yes

Reviewer Expertise:

As a professional with my high academic degree as a Doctor in Management, and my experience in business administration and as a research work evaluator, I can vouch that the research work meets the scientific rigours required by high impact journals.

I confirm that I have read this submission and believe that I have an appropriate level of expertise to confirm that it is of an acceptable scientific standard.

# Reviewer report: Reviewer response for version 1

**DOI:** 10.5256/f1000research.157664.r279219

**Published:** 2024-06-12

**Authors:** Walter Ibarra Fretell

**Affiliations:** 1 Universidad Federico Villarreal, Lima, Peru

First and foremost, I appreciate you considering me for the work review.

 This work is important for both practice and theory in administrative sciences, providing a solid foundation for improving public management and advancing scientific knowledge in this area.

 The use of both qualitative and quantitative methods in the study provides a comprehensive view of administrative management in the studied municipality. This approach is critical since it allowed and will allow future studies to capture perception from a mixed perspective, which is always significant.

 This research is significant for administrative sciences as it provides a rigorous methodology and empirical evidence to help make informed decisions and implement better practices in public management. Furthermore, it enriches scientific literature by encouraging the use of mixed approaches and introducing new conceptual frameworks that promote adaptability and innovation in organizations.

 As a suggestion for the future, a better approach to the conclusions could be placed, although it is evident that there is support for the results, the entrepreneurial part and the opinion of the authors is always important to know the point of view of the researchers.

Is the work clearly and accurately presented and does it cite the current literature?

Yes

If applicable, is the statistical analysis and its interpretation appropriate?

Yes

Are all the source data underlying the results available to ensure full reproducibility?

Yes

Is the study design appropriate and is the work technically sound?

Yes

Are the conclusions drawn adequately supported by the results?

Yes

Are sufficient details of methods and analysis provided to allow replication by others?

Yes

Reviewer Expertise:

Master in Finance and PhD in Accounting

I confirm that I have read this submission and believe that I have an appropriate level of expertise to confirm that it is of an acceptable scientific standard.
